# Multidisciplinary Collaborative Efforts in Craniosynostosis Surgery—A Rural Appalachian Institutional Experience in Patient Care and Outcomes

**DOI:** 10.1155/ijpe/7477204

**Published:** 2025-09-11

**Authors:** Brittany McLay, Anushka Pathak, Erik Olness, Antonio Diaz Perez, Christopher Deskins, Andrew Criser, Hal Meltzer, Sebastian Brooke, Frank Casey, Candy M. Mahle, Pavithra R. Ellison

**Affiliations:** ^1^Department of Anesthesiology, West Virginia University, Morgantown, West Virginia, USA; ^2^Department of Neurosurgery, West Virginia University, Morgantown, West Virginia, USA; ^3^Department of Surgery, West Virginia University, Morgantown, West Virginia, USA; ^4^Department of Pediatrics, West Virginia University, Morgantown, West Virginia, USA; ^5^Department of Nursing, West Virginia University, Morgantown, West Virginia, USA

## Abstract

Craniosynostosis surgery, addressing the premature fusion of cranial sutures, presents a unique challenge requiring standardized care, especially in rural settings, to optimize patient outcomes. This study evaluates surgical outcomes from 2012 to 2021, with a focus on 2020–2021, assessing intraoperative red cell–containing products, blood donor exposures, antifibrinolytic use, and ICU and hospital stays across various hospitals, with emphasis on our Rural Appalachian Institution (RAI). Primary outcomes include perioperative blood transfusion rates and hospital stay duration. Secondary outcomes include antifibrinolytic usage and blood donor exposures. Statistical analyses were performed using ANOVA and chi-square tests where applicable, with a significance threshold of *p* < 0.05. Results indicate that preoperative erythropoietin administration at RAI contributed to reduced perioperative blood transfusions. Increased antifibrinolytic use at RAI underscores efforts to minimize transfusions. Despite variations in ICU and hospital stays, RAI's standardized protocols address regional challenges and highlight the necessity for best practices in craniosynostosis care. Future research should explore the impact of erythropoietin on blood conservation, postoperative dexmedetomidine infusion on recovery, and tranexamic acid on perioperative blood management. This study, while acknowledging limitations, provides essential insights for refining surgical protocols in resource-limited settings.

## 1. Introduction

Craniosynostosis is the premature fusion of cranial sutures due to disrupted signaling in the fibroblast growth factor (FGF) pathway. It affects approximately 1 in 2000–2500 births annually, with a higher prevalence in males [1]. Causes include genetic mutations, environmental factors, and metabolic conditions [2]. The condition leads to abnormal skull morphology, increased intracranial pressure, and potential developmental delays [3]. Diagnosis involves physical examination and imaging, including ultrasound and x-ray [4].

Surgical intervention for craniosynostosis includes open cranial vault remodeling (9–12 months) and endoscopic strip craniectomy (< 4 months), with the former remaining the gold standard [5, 6]. Surgical complications include blood loss, infection, prolonged hospitalization, and intensive care unit (ICU) admissions and postoperative opioid usage [7]. Emerging techniques, such as preoperative erythropoietin administration and intraoperative tranexamic acid use, are aimed at minimizing perioperative blood transfusions [8, 9]. Despite surgical risks, mortality rates remain below 1% [9].

This study is aimed at enhancing surgical outcomes for craniosynostosis patients by benchmarking institutional data against national averages. The primary outcome measure is perioperative blood transfusion rates. Secondary measures include antifibrinolytic use, blood donor exposures, and hospital stay duration. By systematically evaluating these metrics, we seek to identify strategies for optimizing craniosynostosis surgical outcomes, particularly in rural healthcare settings.

## 2. Methods

This retrospective cohort study analyzed perioperative data from craniosynostosis surgeries performed at RAI from 2012 to 2021, with a focused analysis of 2020–2021. Patients were selected based on craniosynostosis diagnosis and surgical intervention. Key variables assessed included intraoperative red cell–containing product usage, blood donor exposures, antifibrinolytic administration, and ICU/hospital stays.

Statistical analysis was conducted using ANOVA for continuous variables and chi-square tests for categorical data, with *p* < 0.05 considered statistically significant. Sample size power analysis was performed to ensure adequate statistical strength.

Preoperative care included erythropoietin (600 units/kg/week for 3 weeks) and nutritional optimization. Intraoperative protocols featured arterial line placement, intraoperative tranexamic acid infusion (10 mg/kg bolus, 10 mg/kg/h maintenance), and blood conservation strategies [10]. Postoperative management involved a standardized pain regimen, dexmedetomidine infusion, and transfusion thresholds set at hemoglobin < 7 g/dL or hemodynamic instability [11]. Data comparisons were made with national databases from the Pediatric Craniofacial Collaborative Group (PCCG).

In Appendix 1, the protocol utilized at RAI for patients who undergo craniosynostosis surgery is presented.

## 3. Results

RAI exhibited a median intraoperative red cell–containing product use of 19.3 mL/kg (IQR 9.8–25.9, *N* = 27), compared to 23.3 mL/kg (IQR 13.7–36.6, *N* = 2444) at all hospitals and 5 mL/kg (IQR 0–10.5, *N* = 950) at the top five hospitals. From 2020 to 2021, RAI reported a median of 0 mL/kg compared to 15.7 mL/kg at all hospitals (Tables 1 and 2).

Blood donor exposures at RAI were higher (median 2, IQR 1–4) than all hospitals (median 1, IQR 1–2), but from 2020 to 2021, RAI reduced exposures to a median of 0.

Antifibrinolytic use was 92.6% at RAI (*N* = 27) versus 82.7% at all hospitals (*N* = 2421) and 100% at the top five hospitals (*N* = 573). From 2020 to 2021, RAI achieved 100% fibrinolytic use compared to 98.8% at all hospitals.

ICU stay duration at RAI was a median of 3 days (IQR 3–4) compared to 2 days (IQR 2–2) at all hospitals. Total hospital stay was a median of 4 days (IQR 4–5) at RAI, similar to the national average (Tables 1 and 2).

## 4. Discussion

Key findings highlight that preoperative erythropoietin administration reduces perioperative blood transfusions. Blood donor exposures were initially higher at RAI but significantly decreased in 2020–2021. Antifibrinolytic use at RAI was consistently high, aligning with national best practices. Length of ICU and hospital stays at RAI was slightly longer, potentially reflecting rural healthcare access challenges.

Compared to other rural institutions, RAI's protocol-driven approach demonstrates improved outcomes in blood conservation. Standardized use of erythropoietin, tranexamic acid, and postoperative dexmedetomidine highlights effective perioperative management. These results emphasize the need for standardized practices across similar institutions. Additionally, these findings underscore the importance of regionalized healthcare strategies tailored to resource-limited settings. Expanding educational initiatives for healthcare providers and enhancing access to preoperative optimization services could further improve patient outcomes. Future efforts should focus on refining perioperative blood management strategies, enhancing multimodal pain control, and leveraging telemedicine for remote perioperative evaluations.

The PCCG has made significant contributions to the understanding and improvement of perioperative care in children undergoing craniofacial surgery, particularly those with craniosynostosis. Through the establishment of the Pediatric Craniofacial Surgery Perioperative Registry (PCSPR), the PCCG has provided valuable insights into perioperative practices, patient outcomes, and opportunities for quality improvement across multiple institutions.

A primary focus of the PCCG has been benchmarking perioperative outcomes and refining management strategies for complex cranial vault reconstruction. PCCG analyzed multicenter data to assess perioperative risks and management strategies, providing critical benchmarks for surgical teams [12]. Additionally, the safety and efficacy of antifibrinolytics in cranial vault reconstructive surgery have been extensively studied, with PCCG reporting on their use and associated outcomes in a large pediatric cohort [13].

A key area of investigation has been comparing surgical techniques and their perioperative impact. Thompson et al. utilized propensity score matching to compare outcomes between endoscopic and open repair for craniosynostosis, contributing to evidence-based surgical decision-making [14]. Similarly, Lang et al. compared spring-mediated cranioplasty with endoscopic strip craniectomy for sagittal craniosynostosis, highlighting key perioperative considerations [15].

The PCCG has also examined perioperative outcomes for specific surgical procedures beyond cranial vault reconstruction. PCCG's multicenter observational study on perioperative management in midface advancement surgery emphasizes anesthesia and blood management strategies [16, 17]. Furthermore, Fernandez et al. investigated transfusion predictors and outcomes in pediatric cranial vault reconstruction, enhancing perioperative blood management protocols [18].

Recognizing the importance of predictive modeling in perioperative care, the PCCG has explored machine learning applications to improve clinical decision-making. Jalali et al. developed a patient-specific prediction model for blood transfusion requirements using registry data, representing a significant advancement in personalized perioperative care [19].

Craniosynostosis surgeries require multidisciplinary coordination, particularly in rural settings where access to specialized care is limited. RAI's standardized pathways have enhanced surgical outcomes, reducing perioperative transfusions and optimizing perioperative management. Future initiatives should focus on refining transfusion strategies, incorporating emerging pharmacologic interventions, and expanding research on best practices for rural healthcare institutions. Enhancing surgical education, telemedicine integration, and regional collaboration will be essential in further improving patient outcomes in underserved communities.

## 5. Conclusion

Craniosynostosis surgeries require multidisciplinary coordination, particularly in rural settings where access to specialized care is limited. RAI's standardized pathways have enhanced surgical outcomes, reducing perioperative transfusions and optimizing perioperative management. Future initiatives should focus on refining transfusion strategies, incorporating emerging pharmacologic interventions, and expanding research on best practices for rural healthcare institutions. Enhancing surgical education, telemedicine integration, and regional collaboration will be essential in further improving patient outcomes in underserved communities.

## Figures and Tables

**Table 1:** Craniofacial surgical outcomes (2012–2021).

**Outcome**	**RAI median (IQR)**	**Top 5 institutions' median (IQR)**	**All institutions' median (IQR)**
Blood transfusions	19.3 (9.8–25.9)	5 (0–10.5)	23.3 (13.7–36.6)
Blood donor exposure	2 (1–4)	1 (1–1)	1 (1–2)
Antifibrinolytic use (%)	92.6	100	82.7
*Length of stay (days)*			
PICU	3 (3–4)	1 (1–1)	2 (2–2)
Total	4 (4–5)	4 (3–4)	4 (4–5)

Abbreviations: IQR, interquartile range; RAI, Rural Appalachian Institution.

**Table 2:** Craniofacial outcomes (2020–2021).

**Outcome**	**RAI**	**Top 5 institutions**	**All institutions**
Blood transfusions (median)	0	5	15.7
Blood donor exposure (median)	0	1	1
Antifibrinolytic use (%)	100	100	98.8
*Length of stay (days)*			
PICU median	2	2	2
PICU (IQR)	2–4	1–2	2–2
Total median	4	3	4
Total (IQR)	4–5	3–4	4–5

Abbreviations: IQR, interquartile range; RAI: Rural Appalachian Institution.

## Data Availability

Institutional data for outcomes and national data for outcomes are available through the Pediatric Craniofacial Collaborative Group registry.

## Appendix 1: Current Perioperative Protocol for Craniosynostosis Surgery at RAI

Preoperative phase: Understanding and standardizing protocols before craniosynostosis surgeries is crucial for optimizing patient outcomes. Given the complexity of the perioperative period, interviews with team members have informed the development of a comprehensive protocol covering the preoperative, intraoperative, and postoperative phases.

1. Neurodevelopmental and ophthalmologic assessment:
  o. Patients undergo a neurodevelopmental evaluation, including an ophthalmologic exam to assess for elevated intracranial pressure and neurodevelopmental delays.
2. Surgical planning:
  o. Multidisciplinary consultations with plastic surgery and neurosurgical teams.
3. Preoperative blood management:
  o. To minimize perioperative blood transfusions, patients receive erythropoietin 6 weeks before surgery (600 units/kg weekly for 3 weeks).
  o. Hemoglobin levels are monitored, aiming for a preoperative target of 13 g/dL.
4. Nutritional assessment:
  o. Given regional food insecurity challenges, all patients are evaluated preoperatively by a nutritionist for optimization.

Intraoperative phase:

1. Presurgical preparations:
  o. Nonanesthetized patients do not undergo further blood draws before surgery.
  o. After anesthesia induction, peripheral IV lines are placed, and a baseline CBC and type and cross are obtained.
2. Airway and hemodynamic monitoring:
  o. Secure nasotracheal intubation.
  o. Placement of an arterial line for continuous blood pressure monitoring and lab draws.
3. Blood management:
  o. Blood products are made available before bone incisions.
  o. Autologous blood transfusion and whole blood are preferred if needed intraoperatively.
  o. Tranexamic acid (bolus 10 mg/kg, infusion 10 mg/kg/h) is administered to minimize bleeding.
  o. Mannitol (0.5 g/kg) and dexamethasone (0.5 mg/kg) are given as needed to reduce intracranial pressure before craniotomy.
  o. Dexmedetomidine infusion (0.5–1 mg/kg/h) is initiated and continued postoperatively.
4. Extubation and transfer:
  o. Stable patients meeting criteria are extubated in the operating room.
  o. Patients are transported to the pediatric intensive care unit (PICU) with the surgical, anesthesia, and nursing teams.

Postoperative phase:

1. Handoff and communication:
  o. A structured handoff includes surgical descriptions, concerns, and plans.
  o. The anesthesia team provides details on the patient's medical history, airway management, hemodynamics, blood products, and expected complications.
2. Patient monitoring:
  o. Close monitoring for hemodynamic instability, cerebrospinal fluid leaks, pain control, nausea/vomiting, and signs of infection.
3. Laboratory and transfusion protocol:
  o. A structured lab schedule limits blood draws:
    ▪ On arrival to PICU
    ▪ 2000 on Postoperative Day 0
    ▪ Morning of Postoperative Day 1
  o. A transfusion threshold is set at hemoglobin < 7 g/dL or unexplained hemodynamic instability.
4. Pain management:
  o. A multimodal regimen minimizes opioid use:
    ▪ Scheduled IV acetaminophen and as-needed morphine.
    ▪ On Postoperative Day 1, scheduled ibuprofen/ketorolac is introduced, and acetaminophen is transitioned to oral if tolerated.
    ▪ Scheduled ondansetron for the first 48 h.
    ▪ Dexmedetomidine infusion continued until Postoperative Day 1.
5. Recovery and discharge:
  o. Patients are transferred from PICU to the floor on Postoperative Day 1.
  o. Average discharge occurs on Postoperative Day 2 or 3.

Outcome monitoring: Quarterly data collection from the Pediatric Craniofacial Collaborative Group includes comparisons among the top five hospitals nationwide, all hospitals, and our Rural Appalachian Institution (RAI). Data from 2012 to 2021, with a focus on 2020–2021, was analyzed to assess protocol effectiveness. Outcomes examined include the following:

• Intraoperative red cell–containing product (IRCCP):
  o. Calculated as the total amount of packed red blood cells (milliliter) + whole blood (milliliter) + reconstituted blood (milliliter), divided by the patient's weight (kilogram).
• Blood donor exposures (BDE):
  o. Intraoperative BDE: Number of discrete intraoperative transfusions (PRBCs, FFP, platelets, cryoprecipitate, whole blood) minus FFP units from the same PRBC donor.
  o. Postoperative BDE: Total number of discrete postoperative transfusions minus remaining portions of intraoperative transfusions.
• ICU and hospital length of stay:
  o. Total days in PICU recorded as any portion of a calendar day spent in the PICU.

By continuously refining this protocol and assessing its effectiveness, we aim to optimize perioperative care and improve surgical outcomes for patients undergoing craniosynostosis repair, particularly in rural healthcare settings.
